# Risk factor analysis for extended-spectrum β-lactamase-producing *Enterobacter cloacae* bloodstream infections in central Taiwan

**DOI:** 10.1186/1471-2334-13-417

**Published:** 2013-09-08

**Authors:** Chang-Hua Chen, Chieh-Chen Huang

**Affiliations:** 1 Division of Infectious Diseases, Department of Internal Medicine, Changhua Christian Hospital, 135 Nanshiao street, Changhua 06, Taiwan; 2 Department of Nursing, College of Medicine & Nursing, Hung Kuang University, No. 1018, Sec. 6, Taiwan Boulevard, Shalu District, Taichung 43302, Taiwan; 3 College of Life Science, National Chung Hsing University, No. 250 Kuo-Kuang Road, Taichung 40227, Taiwan

**Keywords:** *Enterobacter cloacae*, Class 1 integron, Extended-Spectrum β-Lactamase, Mortality, Risk factors, Bloodstream infections

## Abstract

**Background:**

*Enterobacter cloacae (E.cloacae)* bloodstream infection (EcBSI) is an important cause of morbidity and mortality, with an increasing incidence in our hospital. We wanted to elucidate the risk factors of mortality among patients with ESBL-positive EcBSI in central Taiwan.

**Methods:**

We ordered the clinical and microbiological data of cases with diagnosis of EcBSI, and analyzed the isolates by using antibiotyping, detection of ESBL, detection of class 1 integron and genomic fingerprinting by pulsed-field gel electrophoresis (PFGE).

**Results:**

Seventy episodes of EcBSI from 70 patients (56 hospital-acquired infections) were enrolled. Significant differences were found between ESBL-positive and ESBL-negative isolates with regard to risk factors, including the diseases severity (*p* = 0.03), category of health care-associated infection (*p* = 0.04), prior use of antibiotics (*p* = 0.023), and prior use of a ventilator (*p* = 0.037). A significant difference in mortality between two groups (*p* = 0.004) was determined using the chi-square test, and a trend in mortality between two groups (*p* = 0.006, OR = 4.750, 95% C.I.=1.573-14.344) was determined using univariate logistic regression analysis. The predominant clone in ESBL-positive strains was associated with a higher mortality rate but not with the presence of the integron.

**Conclusions:**

The study disclosed four types of clinical characteristics to obtain ESBL-positive EcBSI, and there was a trend in mortality too. We suggested the need to review antibiotic prescription practices, and the possible need to consider ESBL-positive strains in empirical treatment of bloodstream infection.

## Background

*Enterobacter cloacae (E. cloacae)* is the most frequently isolated species of *Enterobacter*, which is a genus within the family Enterobacteriaceae [1] that causes infections in hospitalized and debilitated patients and has emerged as an important bacterial pathogen in recent years [2]. *E. cloacae* bloodstream infection (EcBSI) is an important cause of morbidity and mortality in both developing and developed countries, especially Taiwan [3,4].

In the last 20 years, extended-spectrum β-lactamases (ESBLs) have attained great medical importance [5]. With regard to the expression mechanism, bacterial class 1 integrons, such as *IntI1* integron, contain a site-specific recombination system able to capture and express genes as gene cassettes [6], and most cassette-integrated β-lactamase genes, such as *bla*_VEB-1_ and *bla*_GES-1_, encode β-lactamases [7,8]. Jiang et al. found that 12 clonally related *E. cloacae* isolates from the China possessed a novel *bla*_VEB-3_-type beta-lactamase that was located in an integron [9], and some studies have reported that Taiwan *E. cloacae* strains contain the *bla*_Imp-8_ and *bla*_SHV-12_ genes [3,4].

The isolation rate of *E. cloacae* has increased since 1995 at the hospital in Taiwan evaluated in the present study. Preliminary findings suggested an increased prevalence of *E. cloacae* that was associated with high mortality from *E. cloacae* infection and high prevalence of the *IntI1* integron; thus, in the present study, we surveyed the ESBLs and integrons of *E. cloacae*. We collected the clinical and microbiological data of cases with a diagnosis of EcBSI and analyzed the isolates by antibiotyping, detection of ESBLs, detection of the class 1 integron and genomic fingerprinting by pulsed-field gel electrophoresis (PFGE). The goal of the study was to elucidate the risk factors for mortality among patients with ESBL-positive EcBSI in central Taiwan.

## Methods

### Hospital setting and data collection

The study hospital is an 1800-bed teaching hospital located in the center of Changhua in central Taiwan. A cross-sectional retrospective study of EcBSI was conducted by the division of infectious diseases of the study hospital from January 1, 2001 through December 31, 2003. The clinical details were recorded, and any mention of risk factors (only those predating the infection) was recorded. The severity of the underlying disease was categorized using the criteria of McCabe & Jackson [10]. The source of infection was determined according to clinical and microbiological evidence and categorized according to its site of origination. All patients with documented EcBSI at the hospital were enrolled during the study period. Repeated isolation of *E. cloacae* within a two-week period was defined as one episode of EcBSI. Only the first isolate obtained from each patient was studied. The exclusion criteria included inadequate data and misidentification. The ESBL-positive and ESBL-negative groups were defined according to the microbiological reports. The integron groups were defined according to the results of *IntI1* gene detection. Health care-associated infection (HAI) was defined according to criteria of the Center of Disease Control and Prevention (CDC) [11]; in particular, HAI was defined when all CDC-defined conditions for site-specific infection were first present together on or after the third day of admission in the hospital.

### Ethical approval

The collection of these data as described above was approved by the Ethics Committee of Changhua Christian Hospital (the reference number is CCH 121,112).

### Microbiological investigation

Blood cultures were performed for every patient with suspected sepsis using the BACTEC NR-860 system (Becton Dickinson Diagnostic Instrument Systems, Franklin Lake, NJ). *E. cloacae* was presumptively identified using colonial morphology, Gram staining and routine biochemical reactions, and the identification was confirmed using the API-20NE kit (Bio Merieux Vitek, Hazelwood, Mo). Routine antibiotic sensitivity testing using β-lactams (amoxicillin-clavulanate, piperacillin-tazobactam, cefazolin, cefuroxime, flomoxef, cefotaxime, ceftriaxone, ceftazidime, cefepime and imipenem-cilastatin), aminoglycosides (amikacin, gentamicin and tobramycin), fluoroquinolones (ciprofloxacins and ofloxacin) and trimethoprim-sulfamethoxazole was performed using the disk diffusion method (BBL, Sensi-Disc; Becton Dickinson, Cockeysville, MD), according to the guidelines of the Clinical and Laboratory Standards Institute (CLSI) [12]. Antimicrobial susceptibility testing for confirmation and detection of ESBLs was performed on the Vitek-2 System (Biomerieux, Hazlewood, Mo.), which has been shown to be 99% sensitive and specific for the detection of ESBLs [13]*.*

### Molecular detection of ***IntI***1

Polymerase chain reaction (PCR) was performed for all *E. cloacae* for detection of IntI*1.* Koeleman’s previously described method was used to amplify the target genes [14]. *IntI1* F (5′-CAGTGGACATAAGCCTGTTC-3′) and R (5′-CCCGAGGCATAGACTGTA-3′) primers were used to detect the *IntI1* gene. PCR amplification was conducted in 20-μL volumes containing 0.25 μL template DNA, 0.025 mM deoxynucleoside triphosphate (dNTP), 1 μL 10 K PCR buffer, 1U *Taq* polymerase (Perkin-Elmer Applied Biosystems, Foster City, CA), 1.5 mM MgCl_2_ and each primer at a concentration of 0.5 μM. PCR amplification was performed using a PTC-200 Gradient Peltier Thermal Cycler (MJ Research, Waltham, MA). The amplification products were resolved by electrophoresis at 125 V for 1 h on a 1.5% agarose gel in 0.5 × Tris-borate-EDTA buffer containing ethidium bromide, followed by visualization under UV light. All PCR amplifications were performed in duplicate.

### PFGE analysis

Genomic DNA isolated from *E. cloacae* was digested using *Xba*I (Takara Bio Co., Japan), and the standard PFGE protocol for *E. cloacae* was followed according to Fernández’s procedure [15]. Briefly, *E. cloacae* isolates were grown on blood agar plates incubated in 5% CO_2_ at 35°C for 16 to 24 h. Plug slices with a width of 2 mm were digested using 20 U of *Xba*I. The DNA fragments were then separated in 1% Seakem Gold agarose gel (FMC BioProducts) at 14°C using a Bio-Rad CHEF DRIII (Bio-Rad Laboratories) in 0.5 × Tris-borate-EDTA (TBE; pH 8) at a 120° fixed angle using a fixed voltage (6 V/cm) and pulse time intervals from 2.2 to 54.2 s for 22 h. *Xba*I-digested genomic DNA fragments of *S. enterica* ser. Braenderup H9812 were used as reference size markers. After staining and destaining of the gel, it was exposed on a LJV transilluminator, and the image was captured digitally using a gel documentation system (Alphalmager 2000; Alpha Innotech Corporation, San Leandro, Calif.). The interpretation criteria were described previously by Tenover [16].

### Statistical analysis

Either the chi-squared test or Fisher’s exact test was used for analysis. The results were considered significant when *P* < 0.05. All data were analyzed using SPSS software v10.0.

## Results

Eighty patients with a diagnosis of EcBSI were identified during the study period, but 10 patients were excluded because of inadequate data. Seventy-eight *E. cloacae* isolates were recovered from the remaining 70 patients, and only the first isolate obtained from each patient was studied. Fifty-six of the EcBSI isolates were identified as HAI, and the remainder was undefined because the patients were referred from either a local hospital or the community. Overall, 20 of 70 *E. cloacae* isolates possessed ESBL, and 49 of them possessed the *IntI1* gene.

The demographics of the 20 patients with ESBL-positive EcBSI are presented in Additional file 1. The male to female ratio was 8:12, and the age range was 0–82 years. Fourteen of the 20 patients with ESBL-positive EcBSI had fever. The initial presentation of the 20 patients included abdominal pain(5 patients), shortness of breath(4), conscious disturbance(4), hematuria(2), deafness(1), flank pain(1), chest pain(1), dysuria(1), and limb weakness(1). Three of them developed shock at initial presentation. Only three of them had received effective empirical antibiotic treatment at the early stage of EcBSI. Twelve of the 20 patients (60%) died. Nine of the 12 deaths directly resulted from the EcBSI (Additional file 1).

The results of the risk factor analysis are listed in Table 1. There was a significant difference between the ESBL-positive and ESBL-negative groups with regard to disease severity (*p* = 0.03), category of HAI (*p* = 0.04), prior use of antibiotics (*p* = 0.023) and prior use of a ventilator (*p* = 0.037). Table 2 shows the analysis of the clinical presentation in patients with ESBL-positive and ESBL-negative isolates. In patients with ESBL-negative isolates, the male to female ratio was 29:21. Overall, urinary tract infection was the only factor significantly different between patients with ESBL-positive isolates and those with ESBL-negative isolates, either in clinical presentation or microbiological characteristics. As shown in Table 3, there was a significant difference in mortality between patients with ESBL-positive isolates and those with ESBL-negative isolates (*p* = 0.004), and mortality tended to be different between patients with ESBL-positive isolates and those with ESBL-negative isolates (*p* = 0.006, OR = 4.750, 95% C.I.=1.573-14.344) in the univariate logistic regression analysis. Forty-nine of the 70 isolates (70%) carried the *IntI1* gene, and 18 of the 49 patients (36.7%) with these isolates died. There was no significant difference between patients with integron-positive *E. cloacae* and those with integron-negative isolates (Table 3).

**Table 1:** Analysis of the risk factors between 20 patients with ESBL-positive EcBSI and 50 patients with ESBL-negative EcBSI

		**ESBL-negative *E. cloacae***	**ESBL-positive *E. cloacae***	**p-value**
		**Total number**	**Total number**	
Underlying disease				
Diabetes mellitus		14	6	
Liver cirrhosis		5	2	
Cancer		12	5	
Uremia		3	1	
McCabe category	A	19	5	0.03
	B	19	5	
	C	12	10	
Belong to Health care-associated infection		36	20	0.04
Invasive procedures before *E. cloacae* infection		4	20	0.481
Chemotherapy before *E. cloacae* infection		1	2	0.482
Operation before *E. cloacae* infection		15	1	0.481
Usage of central venous catheter before *E. cloacae* infection		50	20	0.464
Usage of peripheral catheter before *E. cloacae* infection		50	20	0.301
Usage of arterial catheter before *E. cloacae* infection		24	18	0.857
Usage of Swan-Guan catheter before *E. cloacae* infection		4	3	0.481
Usage of total parenteral nutrition before *E. cloacae* infection		35	17	0.65
Usage of antibiotics before *E. cloacae* infection		46	17	0.023
Usage of ventilator before *E. cloacae* infection		47	19	0.037
Usage of Foley catheter		42	20	0.657

P-value by chi-squared test or Fisher’s exact test when appropriate.

**Table 2:** Clinical presentation between ESBL-positive EcBSI and ESBL-negative EcBSI

		**ESBL(−)**						**ESBL(+)**						**P-value**
			**Integron(−)**		**Integron(+)**				**Integron(−)**		**Integron(+)**			
		**Total**	**N**	**%**	**N**	**%**	**P-value**	**Total**	**N**	**%**	**N**	**%**	**P-value**	
Sex	Male	29	9	31.0	20	69.0	0.851	8	2	25.0	6	75.0	1.000	1.000
	Female	21	6	28.6	15	71.4		12	4	33.3	8	66.7		1.000
Previous antibiotics at the empirical stage before	No	24	1	5.0	23	95.0	1.000	17	7	41.2	10	58.8	1.000	0.023
*E. cloacae* infection	Yes	26	16	61.0	10	39.0		3	1	33.3	2	66.7		
Prognosis	Survival	38	18	47.4	20	52.6	0.874	8	2	25.0	6	75.0	1.000	0.435
	Expired	12	6	50.0	6	50.0		12	2	16.7	10	83.3		0.193
Type of infection	Primary	1	0	0.0	1	100.0	1.000	5	2	0.0	3	100.0	1.000	
	Secondary	49	9	18.4	40	81.6		15	4	21.1	11	78.9		1.000
Secondary infection (multiple)	Total	49	19		30			15	5		10			
	Respiratory tract	30	10	33.3	20	66.7	0.326	3	1	33.3	2	66.7	0.370	1.000
	Urinary tract	12	2	16.7	10	83.3	0.095	6	1		5		0.023	1.000
	Catheter-related	2	0	0.0	2	100.0	0.515	0	0	0.0	0	100.0	1.000	
	Intra-abdominal infection	9	2	22.2	7	77.8	0.451	6	0	0.0	6	100.0	0.474	1.000
	CNS infection	3	1	33.3	2	66.7	1.000	0	0	0.0	0	0.0		
	Others	3	1	33.3	2	66.7	1.000	0	0	0.0	0	00	0.474	1.000

P-value by chi-squared test or Fisher’s exact test when appropriate.

**Table 3:** Univariate logistic regression analysis for mortality between ESBL-positive EcBSI and ESBL-negative EcBSI

		**Total**	**Expired**		P-value*	**Univariate logistic regression analysis**						
			**N**	**%**		**B**	**SE**	**OR**	**95% CI for OR**			**P-value**
Overall	ESBL(−)	50	12	24.0	0.004	1.558	0.564	1.000	1.573	-	14.344	0.006
	ESBL(+)	20	12	60.0				4.750				
ESBL(−)	Integron(−)	24	6	25.0	0.874	−0.105	0.662	1.000	0.246	-	3.297	0.874
	Integron(+)	26	6	23.1				0.900				
ESBL(+)	Integron(−)	4	2	50.0	1.000	0.511	1.125	1.000	0.184	-	15.130	0.650
	Integron(+)	16	10	62.5				1.667				

*:by chi-squared test or Fisher’s exact test when appropriate.

OR: odds ratio.

**Figure 1:**
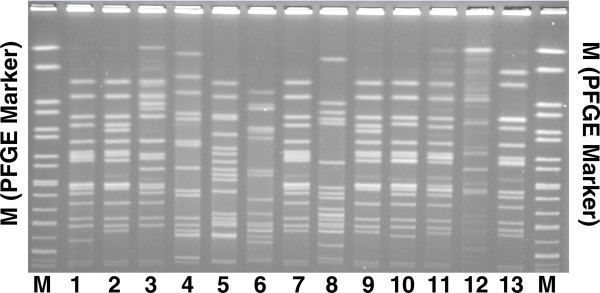
**PFGE fingerprints of 11 ESBL(+) *E. cloacae *isolates and 2 ESBL(−) *E. cloacae *isolates after digestion with the *Xba*I restriction enzyme.** Lane 1 to lane 10, ESBL(+) *E. cloacae* isolates; lane 11 to lane 13, ESBL(−)*E. cloacae* isolates; lane M, molecular size markers (PFGE marker, *S. enterica* ser.*Braenderup* H9812).

All 13 strains tested (11 ESBL-positive and 2 ESBL-negative) were typeable. Ten ESBL-positive isolates from expired patients and one each from an ESBL-positive isolate from a survivor, an ESBL-negative isolate from an expired patient and an ESBL-negative from a survivor were analyzed to investigate differences in clinical outcome between ESBL-positive and ESBL-negative strains and to establish the major clones. The fingerprints generated using *Xba*I restriction endonucleases are shown in Figure 1. There was no evidence of clonal dissemination during the three years of the study. The predominant clone was found in most of the ESBL-positive strains.

## Discussion

*E. cloacae* bloodstream infection is an important cause of morbidity and mortality in both developing and developed countries. In our hospital, an increasing incidence and a high crude mortality rate (24/70, 34.3%) led us to investigate the epidemiology of this infection, particularly with regard to ESBL, and to determine whether clonal spreading occurred.

In a previous epidemiological study of *E. cloacae* infection, Liu showed that central venous catheterization and mechanical ventilation increased the relative risk for nosocomial *E. cloacae* infection; that age and mechanical ventilation were risk factors for multiresistant *E. cloacae* infection; and that mortality was associated with multiresistant isolates and polymicrobial infection [4]. These risk factors were similar to those identified in our study, which included disease severity (*p* = 0.03), category of hospital-acquired infection (*p* = 0.04), prior use of antibiotics (*p* = 0.023) and prior use of a ventilator (*p* = 0.037). We also demonstrate that mortality tended to be different (*p* = 0.006, OR = 4.750, 95% C.I.=1.573-14.344) between infections with ESBL-positive *E. cloacae* and those with ESBL-negative *E. cloacae*.

ESBL-expressing *E. cloacae* present a challenge for clinical microbiologists and clinicians because of the high morbidity and mortality associated with infection. In our study, some ESBL-positive stains were sensitive to imipenem-cilstatin. However, there have already been reports of therapeutic failures of imipenem-cilstatin in strains that produce multiple β-lactamases [17]. The high mortality rate observed for ESBL-positive EcBSI likely results from this difficulty in treatment. In the present study, there was a significant difference in mortality between patients with ESBL-positive EcBSI and those with ESBL-negative EcBSI (*p* = 0.004), and mortality tended to be different between patients with ESBL-positive EcBSI and those with ESBL-negative EcBSI (*p* = 0.006, OR = 4.750, 95% C.I. = 1.573-14.344) in a univariate logistic regression analysis. These findings may be attributed to the fact that 17 of the 20 patients with ESBL-positive EcBSI did not receive effective empirical antibiotic treatment, whereas 46 of the 50 patients with ESBL-negative isolates did receive effect antibiotic treatment.

This is the first study to report the relationship between ESBLs and an integron in *E. cloacae.* There was no significant difference between patients with integron-positive EcBSI and those with integron-negative EcBSI. However, mortality tended to be different between patients with ESBL-positive EcBSI and those with ESBL-negative EcBSI (*p* = 0.004). The development and spread of ESBL-positive EcBSI has been suggested to be caused by the overuse of expanded-spectrum cephalosporins in the hospital setting [3]. We agree with this mechanism but also consider that the epidemiology of ESBL-positive EcBSI requires further investigation. We observed a high prevalence (70%) of the integron, which also raises the question of whether antibiotic selective pressure in hospitals in Taiwan may have led to the dissemination of this integron, which contains an ESBL-carrying cassette. β-lactam, aminoglycoside and sulfonamide resistance genes are all associated with class 1 integrons and may enhance the dissemination of these integrons. Similar to Severino [18], we suggest that integron detection should be used for the study of molecular epidemiology in hospital environments, particularly for the detection of possible cross-infection cases and estimation of accumulative antibiotic selective pressure.

The increased prevalence of *E. cloacae* and its association with high EcBSI mortality and high prevalence of *IntI1* motivated us to perform PFGE, which is an auxiliary investigation method for *E. cloacae* isolates that has high reproducibility and high discriminatory power and is thus regarded as the “gold standard” for defining a clone in various nosocomial bacterial populations [2,19]. The predominant clone was associated with increased mortality that was most likely related to ESBL production; however, the predominant clone was not associated with the presence of the integron. Clonal spread was not observed, and the class 1 integron was not a major factor in the increased incidence of ESBL-positive *E. cloacae* in the study hospital. Ho et al. used PFGE genotyping to show that seven ESBL- positive *E. hormaechei* isolates were unrelated [20]. Our results are similar and show a lack of clonal outbreak during the three years of the study. The predominant clone seemed to be associated with increased mortality and ESBL expression but not with the presence of the integron.

Although the bacterial isolates were collected between 2001 and 2003, as the clinical pattern of EcBSI did not show obvious changes in our institute within the last decade (2002–2012), the results are still relevant for clinical application at the present time and agree with the findings of Freeam [21].

## Conclusions

This study identified four types of clinical characteristics for ESBL-positive EcBSI, and increased mortality was associated with the presence of ESBL, which most likely resulted from inappropriate empirical treatment. We suggest that it is necessary to review antibiotic prescription practices and possibly consider ESBL-positive strains in the empirical treatment of bloodstream infections.

## Competing interests

Both authors declare that they have no competing interests.

## Authors’ contributions

Both CHC and CCH designed and performed this study. CCH analyzed the data regarding the infectious diseases and wrote the manuscript. Both authors read and approved the final manuscript.

## Pre-publication history

The pre-publication history for this paper can be accessed here:

http://www.biomedcentral.com/1471-2334/13/417/prepub

## Supplementary Material

Additional file 1 Basic information of 20 patients with ESBL-positive EcBSI. Click here for file
