# Supplementary material for: Risk factor analysis for extended-spectrum β-lactamase-producing Enterobacter cloacae bloodstream infections in central Taiwan
Source: BMC Infect Dis. 2013 Sep 8;13:417. doi: 10.1186/1471-2334-13-417 (PMC3846452; doi:10.1186/1471-2334-13-417)
Supplement: Additional file 1 — Basic information of 20 patients with ESBL-positive EcBSI. [file 1471-2334-13-417-S1.doc]

Supplement 1 Basic information of 20 patients with ESBL-positive EcBSI

| Item | Description |
| --- | --- |
| General information |  |
| Age | 1-88 years-old |
| Gender ( male ) | 9 patients |
| Times of previous admission before EcBSI | 0-3 times |
| Duration of chief complain before EcBSI | 1-7 days |
| Underlying diseases |  |
| Diabetes mellitus | 6 patients |
| Cancer | 5 patients |
| Congestive heart failure | 2 patients |
| Stone at urinary tract | 2 patient |
| Stone at biliary tract | 1 patients |
| Liver cirrhosis | 1 patient |
| Uremia | 1 patient |
| Initial clinical manifestations |  |
| Fever | 14 patients |
| Shock | 3 patients |
| Route of entry |  |
| Urinary tract | 6 patients |
| Biliary tract | 6 patients |
| Primary | 5 patients |
| Respiratory tract | 3 patients |
| Major antimicrobial therapy |  |
| Imipenem-cilastatin | 13 patients |
| Piperacillin-tazobactam | 2 patients |
| Cefepime | 2 patients |
| Ceftizadime | 1 patient |
| Ampicillin-sulbactam | 1 patient |
| Cefuroxime | 1 patient |
| Outcome |  |
| Survival | 8 patients |
| Expired | 12 patient |
